# Upcycling Spent Graphite Anodes into Bifunctional Photothermal Catalysts for Efficient PET Chemical Recycling

**DOI:** 10.1002/advs.202510772

**Published:** 2025-09-17

**Authors:** Yeping Xie, Mingle Qiu, Binglei Jiao, Panpan Xu, Muhan Cao, Qiao Zhang, Jinxing Chen

**Affiliations:** ^1^ State Key Laboratory of Bioinspired Interfacial Materials Science Institute of Functional Nano & Soft Materials (FUNSOM) Soochow University Suzhou 215123 P. R. China; ^2^ Institute of Functional Nano & Soft Materials (FUNSOM) Jiangsu Key Laboratory of Advanced Negative Carbon Technologies Soochow University Suzhou 215123 P. R. China; ^3^ Soochow Institute for Energy and Materials Innovations College of Energy Soochow University Suzhou 215006 P. R. China

**Keywords:** graphite anode, lithium batteries, photothermal catalysis, plastic pollution, polyester recycling

## Abstract

The economic recovery of anodes in lithium‐ion batteries remains challenging due to their low value. Here, the study presents a cross‐sector battery‐plastic co‐upcycling strategy that transforms spent graphite anodes into bifunctional photothermal catalysts for efficient Polyethylene terephthalate (PET) depolymerization. Upon reaction with ethylene glycol (EG), lithiated graphite spontaneously enables copper foil detachment, graphite regeneration, and in situ formation of organolithium species (C_2_H_4_O_2_Li_2‐x_H_x_). The resulting catalyst system achieves 95% PET conversion and 64.6% BHET yield within 15 minutes under 0.71 W/cm^2^ sunlight. Mechanistically, a synergistic effect between solid electrolyte interphase (SEI)‐derived Li_2_CO_3_/Li_2_O and organolithium intermediates significantly accelerates glycolysis. Techno‐economic modeling for a 90 000 ton/year facility reveals a minimum selling price of $0.956/kg for BHET and annual energy savings of 5.039 × 10^11^ kJ. This work highlights a scalable, low‐cost approach to integrate battery and plastic waste recycling, offering a new paradigm for sustainable urban mining and circular polymer economy.

## Graphical abstract

Waste graphite anodes from spent lithium‐ion batteries serve as a sustainable lithium source for synthesizing lithium ethylene glycolate. This reactive species efficiently catalyzes PET depolymerization under mild conditions, achieving rapid and selective monomer recovery. The synergistic co‐recycling strategy that couples battery and plastic waste advances circular chemistry.

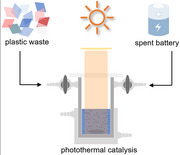

## 1 Introduction

Lithium‐ion batteries (LIBs) have become the cornerstone of modern energy storage systems due to their high energy density, with the global market projected to exceed USD 40 billion by 2030.^[^ ^1^, ^2^ ^]^ However, the widespread adoption of LIBs inevitably leads to a surge in retired batteries. According to the International Energy Agency (IEA), cumulative lithium battery waste is estimated to reach 370 million metric tons globally by 2030.^[^ ^3^ ^]^ Without efficient recycling infrastructure, these end‐of‐life batteries pose significant environmental risks.

Current LIB recyclings technologies primarily focus on recovering high‐value metals (e.g., Co, Ni, Li) from cathode materials. Pyrometallurgical (> 1500 °C calcination) and hydrometallurgical (acid leaching‐precipitation) processes have achieved industrial‐scale metal recovery from ternary NMC LIBs, with overall metal recovery rates exceeding 95%.^[^ ^4^, ^5^ ^]^ Nevertheless, these methods exhibit insufficient economic viability for low‐value cathodes such as LiFePO_4_ (LFP) and LiMn_2_O_4_ (LMO).^[^ ^6^ ^]^ Taking LFP as an example, the recycled FePO_4_ component holds a marginal value of USD 10–15/kg, which fails to offset processing costs.^[^ ^7^, ^8^ ^]^ Although direct regeneration technologies—employing lithium replenishment and sintering to restore cathode structures—have reduced costs by 30–40% in recent years, their application remains constrained by challenges in battery disassembly complexity and performance degradation of regenerated materials.^[^ ^9^, ^10^ ^]^

**Figure 1:**
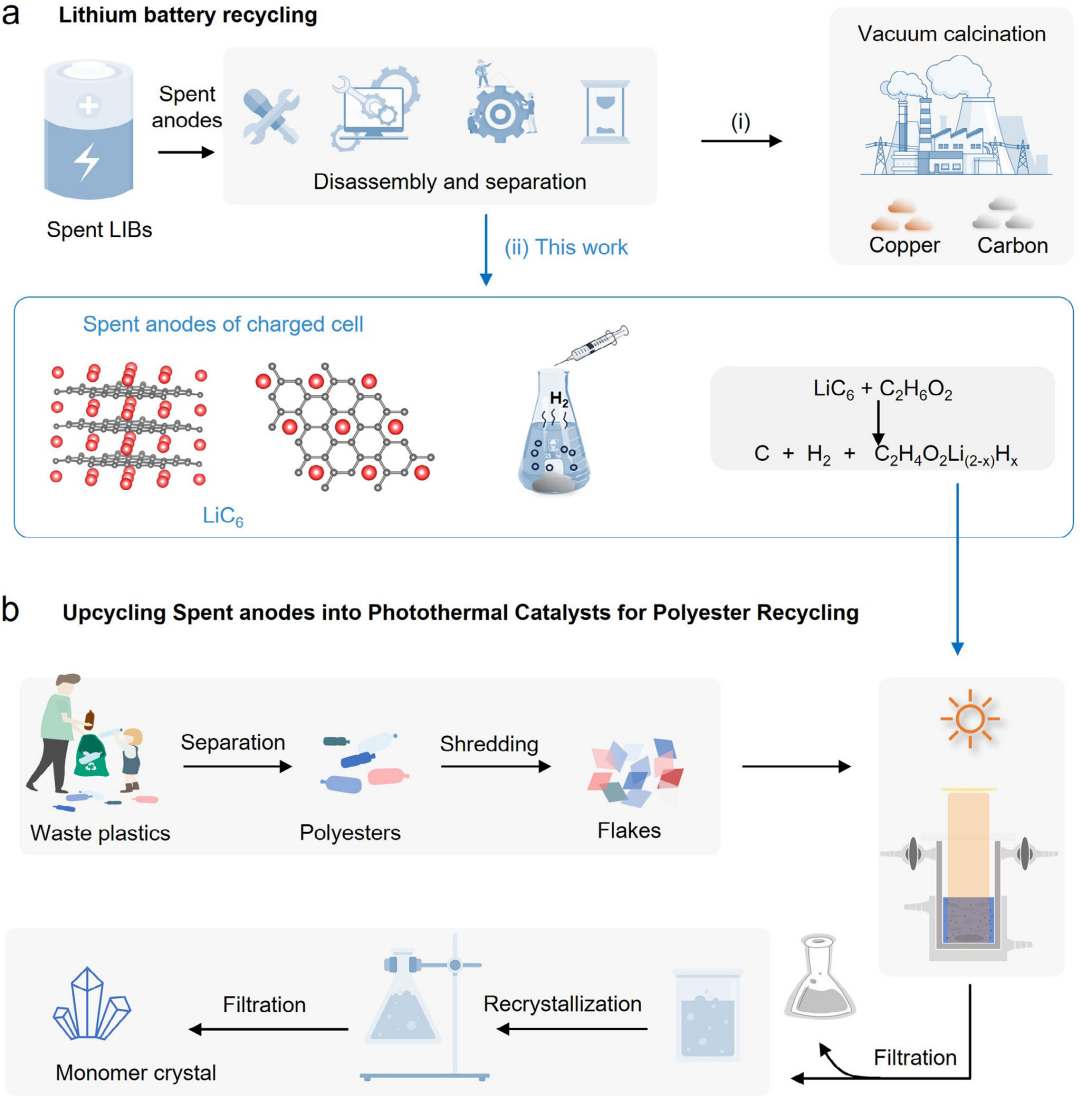
Upcycling of Anode Scrap into Photothermal Catalysts for Polyester Recycling. a) Schematic illustration of strategies for enhancing the residual value of anode materials: (Route 1) direct incineration for the recovery of metallic copper and graphite; (Route 2, this work) upcycling anode scrap into photothermal catalysts. b) Schematic diagram of the process for upgrading anode scrap into photothermal catalysts for the catalytic photothermal depolymerization of waste polyester materials.

In contrast to cathodes, anodes account for 15–20% of battery mass but suffer from a persistent lack of efficient and value‐added recycling strategies. As a result, over 500 000 metric tons of spent graphite from LIBs are landfilled annually worldwide.^[^ ^11^ ^]^ Existing anode recycling processes prioritize copper foil recovery (USD 8.5/kg) while neglecting the resource potential of graphite and residual lithium (2–5 wt.%).^[^ ^12^, ^13^, ^14^ ^]^ More critically, highly reactive lithium species (e.g., LiC_6_) in charged‐state anodes may trigger combustion or hydrogen evolution upon environmental exposure, posing safety hazards.^[^ ^15^ ^]^ As illustrated in **Figure** **1**a, Path I represents the mainstream anode recycling technology, which achieves stepwise recovery of copper foil and graphite through mechanical dismantling–vacuum calcination processes. However, this approach suffers from critical technical bottlenecks, including lithium loss and high energy consumption. Therefore, developing advanced anode recycling protocols is imperative not only for reclaiming strategic resources like copper and graphite but also for mitigating environmental risks and achieving closed‐loop sustainability across the full battery value chain.

In recent years, several efficient strategies have been reported for the direct recovery of active lithium from lithiated graphite anodes. For example, Li et al.,^[^ ^16^ ^]^ demonstrated the preparation of high‐value organolithium reagents from spentLIBs anodes; Guo et al.,^[^ ^17^ ^]^ developed a mild and efficient lithium extraction strategy based on tunable redox pairs in tailor‐made non‐protic solutions composed of polycyclic aromatics and ethers; and Xie et al.,^[^ ^18^ ^]^ employed a polycyclic aromatic–ether system to directly leach active lithium at ambient temperature, which was subsequently utilized in the synthesis of high‐performance LFPcathode materials. These studies not only significantly simplified the lithium recovery process and reduced energy consumption and environmental impact but also demonstrated the potential of recovered lithium for direct application in the synthesis of new materials.

In this work, using retired LFP batteries as a model system, we propose an innovative strategy for value‐added utilization of graphite anodes (Path II in **Figure** 1a), where he reactive lithium species (4.2–5.7 wt.%) in charged‐state graphite spontaneously reacts with EG, enabling three synergistic transformations: (i) Nondestructive copper foil detachment; (ii) Graphite structural regeneration; and (iii) In situ synthesis of organolithium nucleophiles (≈0.12 mol L^−1^). Furthermore, leveraging the regenerated graphite's photothermal properties and the strong nucleophilicity of the derived organolithium reagents, we constructed a solar‐driven photothermal polyester glycolysis system (Figure 1b). This breakthrough achieves co‐recycling of “spent batteries‐waste plastics” through polyethylene terephthalate (PET) depolymerization. Notably, this strategy transcends conventional single‐dimensional recycling paradigms by simultaneously enhancing anode material value and reducing PET chemical recycling energy consumption. This cross‐industrial approach provides transformative insights for addressing dual challenges in battery recycling economics (particularly for low‐value LFP systems) and white pollution mitigation, establishing a prototype for sustainable closed‐loop material cycles.

## 2 Results and Discussion

### 2.1 Upgrading Spent Anodes into Photothermal Catalysts

**Figure 2:**
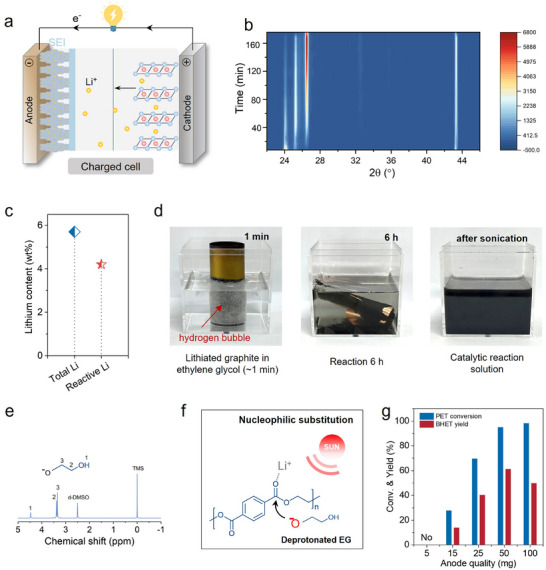
Preparation of Photothermal Catalysts. a) Phase structure of LFP. batteries in the charged state. b) Ex situ XRD patterns of lithiated graphite after varying durations of air exposure. c) Determination of lithium content: total lithium quantified by ICP‐OES analysis; active lithium measured based on the amount of H_2_ evolved from its reaction with water. d) Digital photographs showing each stage in the preparation of organolithium compounds from lithiated graphite. e) H^1^‐NMR spectrum of organolithiums. f) Schematic diagram of the nucleophilic substitution reaction of deprotonated EG. g) Effect of anode mass on PET conversion and BHET yield under photothermal catalysis, reaction conditions: T = 180 °C, m_PET_ = 0.5 g, V_EG_ = 2.5 mL, t = 15 min.

The varying voltages of retired LFP batteries result in compositional complexity, hindering the standardization of model systems. To enhance consistency and reproducibility, all retired LFP batteries were charged to 3.8 V (**Figure** **2**a), enabling the electrochemical lithiation process that drives lithium ions (Li^+^) from the LFP cathode to the graphite anode, forming lithiated graphite (e.g., LiC_6_). This approach ensures uniform lithium content in the anodes across different cells. The batteries were then disassembled in an argon‐filled glovebox to isolate the components. The recovered graphite anode exhibited a golden sheen (Figure S1, Supporting Information), a characteristic appearance of LiC_6_. Residual electrolyte was removed by washing with dimethyl carbonate (DMC), followed by vacuum drying. X‐ray diffraction (XRD) analysis (Figure S2, Supporting Information) revealed a prominent peak at 24.3°, corresponding to the (002) plane of LiC_6_, confirming the stable intercalation of lithium ions within the graphite layers. Upon exposure to air, LiC_6_ underwent a stepwise phase transition: LiC_6_ (24.3°) → LiC_12_ (25.3°) → graphite (26.5°) (Figure 2b), indicating its high chemical reactivity. This dynamic phase evolution not only elucidates the structural transformation pathway of lithiated graphite but also highlights its potential as an active precursor for catalytic applications.

To accurately quantify the active lithium content in spent graphite anodes, we developed a gas‐based quantification method based on hydrolysis. Specifically, 0.1 g of lithiated graphite anode (LiC_6_), charged to 3.8 V, was placed in a sealed 50 mL reactor, followed by the injection of 5 mL deionized water to initiate the displacement reaction (LiC_6_ + H_2_O → LiOH + C + H_2_↑). The generated H_2_ was quantitatively analyzed using gas chromatography (GC‐7900, TCD detector, Figure S3, Supporting Information), allowing back‐calculation of the active lithium content, which was determined to be 4.2 wt.% (Figure 2c). In comparison, the total lithium content measured by inductively coupled plasma optical emission spectroscopy (ICP‐OES) was 5.7  wt.%. The difference (1.5 wt.%) suggests that ≈26.3% of lithium exists in inert forms, such as LiF or Li_2_CO_3_, within the SEI. Mass balance analysis further determined the composition of the anode to be 21.5  wt.% copper, 64.5  wt.% graphite, and 8.3  wt.% SEI components (Figure S4, Supporting Information). To ensure reproducibility, all experiments were conducted in triplicate using anode samples from three different batches of retired LFP batteries, each pre‐charged to 3.8  V.

The obtained lithiated graphite exhibits high reactivity and can directly react with EG to form lithium ethylene glycolate (C_2_H_4_O_2_Li_2‐x_H_x_). Figure S5 (Supporting Information) indicates that hydrogen evolution in the EG system is mild and, under sealed conditions, presents no safety risks, confirming the safety and feasibility of this approach for active lithium recovery. This organolithium compound possesses strong nucleophilicity and shows promise as a catalyst for nucleophilic substitution reactions. The reaction behavior of lithiated graphite with EG is shown in Figure 2d. When 1 g of graphite anode was immersed in 50 mL of EG solution, the displacement reaction was immediately initiated. In the initial stage (0–10 minutes), a large amount of H_2_ was produced; the turbulence from gas bubbles effectively detached the interface between the graphite and copper foil, achieving damage‐free separation of the copper foil. After 6 hours, H_2_ evolution ceased, indicating the active lithium was almost completely consumed. XRD analysis revealed the dynamic evolution of the reaction. At the initial stage, the pattern (Figure S6, Supporting Information) exhibited a prominent LiC_6_ diffraction peak along with weaker LiC_12_ and graphite peaks. As the reaction progressed, the intensity of the LiC_6_ peak gradually decreased, while the LiC_12_ and graphite peaks became progressively stronger. After ≈30 minutes, the LiC_6_ and LiC_12_ peaks completely disappeared, leaving only the characteristic diffraction peaks of graphite. The final product was a mixture of graphite, C_2_H_4_O_2_Li_2‐x_H_x_, and EG. The formation of C_2_H_4_O_2_Li_2‐x_H_x_ was confirmed by ^1^H NMR spectroscopy (Figure 2e), where characteristic peaks appeared at δ = 3.34 and 3.39 ppm, corresponding to the methylene groups coordinated with lithium (‐CH_2_–O–Li) and those adjacent to hydroxyl groups (‐CH_2_–OH), respectively. This was further corroborated by the infrared absorption peak at 1045 cm^−1^ attributed to the C–O–Li stretching vibration (Figure S7, Supporting Information), confirming the formation of the organolithium compound. Laser diffraction analysis showed an average graphite particle size of 15.9 µm (Figure S8a, Supporting Information). Zeta potential measurements yielded a value of –28.2 mV, indicating that the electrostatic repulsion between particles is sufficient to overcome van der Waals forces, ensuring the stability of the suspension.

Photothermal testing (Figure S8b, Supporting Information) was performed under a xenon lamp (CEL‐HXF300‐T3, Beijing China Education Au‐light Co., Ltd.) with a calibrated intensity of 0.6 W/cm^2^, calibrated with a solar power meter (CEL‐NP2000‐2A). The graphite–EG suspension rapidly heated from 25 to 185  °C within 20 min (8  °C min^−1^), owing to graphite's broad‐spectrum light absorption. Figure 2f illustrates the catalytic mechanism of C_2_H_4_O_2_Li_2‐x_H_x_ in PET depolymerization, where deprotonated EG acts as a nucleophile attacking the lithium‐activated carbonyl carbon of PET.^[^ ^19^, ^20^, ^21^, ^22^ ^]^ Optimization experiments (Figure 2g) revealed a loading–activity relationship: using 50 mg anode (2.1 mg active Li) gave the best results—94.9% PET conversion and 61.2% BHET yield. Excess anode loading (>50 mg) led to elevated solution alkalinity, which induced over‐deprotonation of the hydroxyl groups in BHET. The resulting anionic BHET lithium salts exhibit increased solubility in water, making crystallization and recovery more difficult and ultimately reducing the apparent BHET yield. The amount of EG (Figure S9, Supporting Information) significantly affects PET depolymerization efficiency and BHET yield, with an optimal dosage (2.5 mL) ensuring both reaction rate and product recovery; either insufficient or excessive EG reduces the BHET yield. High anode loading severely affects the BHET yield (Figure S10, Supporting Information). However, when using 15 mg of anode, extending the reaction time to 2 hours led to ≈90% PET conversion and over 70% BHET yield (Figure S11, Supporting Information). Additionally, the catalytic activity of fully discharged graphite anodes was examined (Figures S12–S15, Supporting Information). Under the conditions of 50 mg anode loading and a 15‐minute reaction time, the PET conversion was below 5%, indicating that fully discharged graphite anodes exhibit negligible catalytic activity for PET depolymerization.

Regarding the stability of LiC_6_, we evaluated the PET depolymerization performance of the catalyst after different durations of air exposure (10 min, 1 h, 24 h). As shown in Figure S16 (Supporting Information), the sample exposed for 10 min still reacted with EG and released hydrogen, indicating the presence of active lithium species. In contrast, no gas bubbles were observed for the 1 h‐ and 24 h‐exposed samples, suggesting that the active lithium had been largely depleted. This observation aligns with the XRD results in Figure 2b, which show that LiC_6_ rapidly converts to LiC_12_ in air and eventually to graphite. Catalytic performance tests (Figure S17, Supporting Information) further revealed that the 10 min‐exposed sample retained relatively high activity (PET conversion ≈ 80%, BHET yield ≈ 45%). In comparison, the activity of the 1 h‐exposed sample dropped significantly (≈ 55% conversion, ≈ 35% yield), while the 24 h‐exposed sample was nearly inactive (< 10% conversion). These findings indicate that the catalyst can tolerate short‐term air exposure, but prolonged exposure markedly diminishes its performance.

### 2.2 Effect of SEI in Photothermal Catalysis

**Figure 3:**
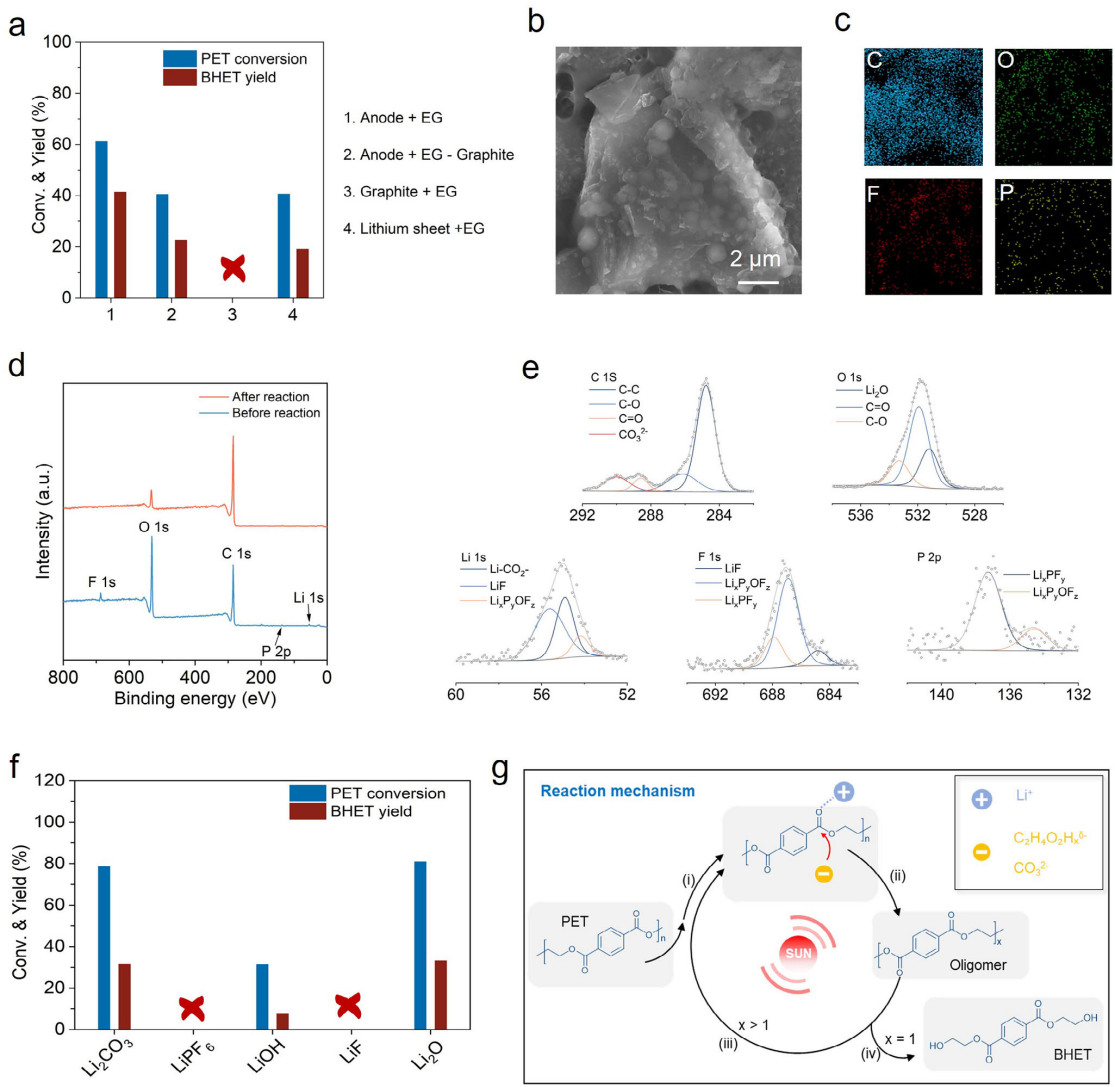
Catalytic performance evaluation and mechanistic investigation. a) Catalytic performance of different material combinations under thermocatalytic conditions, reaction conditions: T = 175 °C, t = 15 min, m_PET_ = 0.5 g, V_EG_ = 2.5 mL, m_anode_ = 50 mg. b) SEM image of the anode surface. c) Elemental distribution mapping of the anode surface. d) XPS survey spectrum of the anode surface. e) High‐resolution XPS spectra of key elements. f) Catalytic performance of various components present in the solid electrolyte interphase (SEI) under identical thermocatalytic conditions, reaction conditions: T = 175 °C, t = 15 min, m_PET_ = 0.5 g, V_EG_ = 2.5 mL. g) Schematic illustration of the PET glycolysis mechanism facilitated by photothermal catalysis.

Lithium metal foil, as a conventional lithium source, faces significant limitations for industrial application of alkoxide‐based catalytic systems due to its high cost and complex purification processes. In contrast, the graphite anode used in this study is directly recovered from spent lithium‐ion batteries and requires only simple pretreatment such as disassembly and cleaning, making the raw material cost nearly negligible. To highlight the clear advantages of the “waste‐to‐treat‐waste” approach, this study systematically compares the catalytic performance of graphite anodes and lithium metal foil in PET glycolysis (**Figure** **3**a). To ensure comparability, the lithium content was strictly controlled to be the same in both catalyst systems (2.85 mg), and the reactions were conducted in a thermostatic oil bath at 175 ± 1 °C to exclude any photothermal effects. Results show that the graphite anode system achieved a PET conversion of 61.2% and a BHET yield of 44.1% within 15 minutes, nearly doubling the performance of the lithium foil system (32.5% and 18.7%, respectively). This difference suggests that, besides the organolithium compounds (C_2_H_4_O_2_Li_2‐x_H_x_), the solid electrolyte interphase (SEI) film on the graphite surface may provide a crucial synergistic catalytic effect. To verify this, graphite particles were removed from the catalytic solution by centrifugation, resulting in PET conversion and BHET yield dropping to 40.5% and 22.6%, respectively—comparable to the lithium foil system—further confirming the key catalytic activity of the graphite surface.

To further elucidate the origin of the catalytic activity, scanning electron microscopy (SEM) was employed to investigate the surface structure and chemical composition of the graphite anodes. SEM imaging (Figure 3b) revealed that the pristine graphite surface was covered by a dense and continuous coating layer. Elemental mapping via energy‐dispersive X‐ray spectroscopy (EDS) (Figure 3c) showed that this layer was enriched in C, O, F, and P. Following the reaction with EG and subsequent water washing, this coating was largely removed (Figure S18a–f, Supporting Information). X‐ray photoelectron spectroscopy (XPS) depth profiling was further conducted to quantify changes in elemental composition. Prior to reaction, the primary elements on the graphite surface were C (51%), O (34.4%), Li (11.5%), F (2.5%), and P (0.6%) (Figure 3d; Figure S19, Supporting Information). High‐resolution spectra (Figure 3e) identified the SEI components as a complex mixture of organic and inorganic lithium salts, including Li_2_O (O 1s, 531.2 eV), Li_2_CO_3_ (C 1s, 290.0 eV), Li_x_PF_y_ (P 2p, 137.27 eV), and LiF (F 1s, 684.80 eV). After reaction and water washing, the atomic percentages of O, F, and P decreased to 11.9%, 0.4%, and 0.4% (Figure S20, Supporting Information), respectively, confirming that the catalytically active SEI components can be effectively removed through aqueous treatment.

To clarify the contribution of individual SEI constituents to catalytic activity, a component‐decoupling strategy was employed. The catalytic performances of representative SEI‐derived compounds—commercial Li_2_CO_3_, Li_2_O, LiPF_6_, and LiF—were compared under identical lithium content (Figure 3f). The results revealed that Li_2_CO_3_ and Li_2_O showed high PET conversion efficiencies (> 75%), while LiPF_6_ and LiF exhibited negligible activity. These findings demonstrate a pronounced synergistic catalytic effect between the SEI‐derived Li_2_CO_3_/Li_2_O and the in situ generated organic lithium species (C_2_H_4_O_2_Li_2‐x_H_x_). Based on these observations, a catalytic mechanism involving the graphite anode was proposed (Figure 3g). Initially, LiC_6_ reacts with EG to form (C_2_H_4_O_2_Li_2‐x_H_x_), while residual Li_2_CO_3_ and Li_2_O in the SEI cooperatively lower the deprotonation energy barrier of EG, thereby increasing the concentration of nucleophilic species. Subsequently, Li⁺ can polarize the carbonyl group in PET molecules, thereby enhancing the electrophilicity of the carbonyl carbon.^[^ ^23^, ^24^, ^25^, ^26^, ^27^ ^]^ Finally, the deprotonated EG species initiates a nucleophilic attack on the activated carbonyl group, leading to ester bond cleavage, ultimately producing the BHET monomer.

### 2.3 Demonstrations

**Figure 4:**
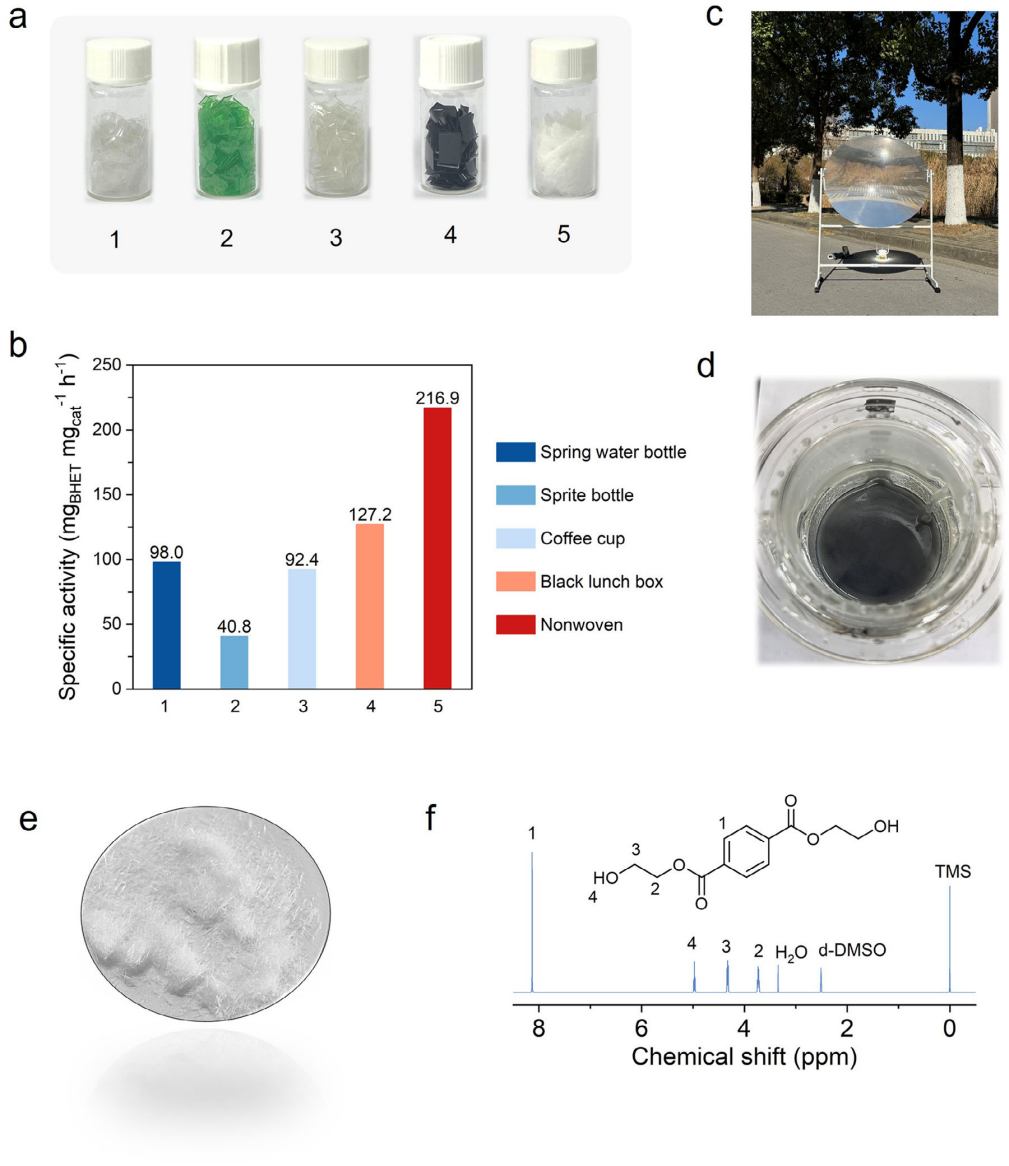
Demonstration of photothermal catalysis‐driven chemical recycling of polyesters and evaluation of energy consumption. a) Images of different post‐consumer polyesters. b) Glycolysis activity of different post‐consumer polyesters. c) Portable outdoor reaction platform used under the following conditions: T = 200 °C, t = 15 min, m_PET_ = 20 g, V_EG_ = 100 mL, m_anode_ = 2 g. d) The state of the system after the outdoor reaction completed. e) Digital image of 17.1 g of BHET. f) ^1^H NMR spectra of produced BHET.

Evaluating the catalytic depolymerization performance of real‐world PET waste is essential for promoting the industrial application of photothermal catalytic technologies.^[^ ^28^, ^29^, ^30^ ^]^ In this study, five representative types of post‐consumer PET waste were selected as model feedstocks: mineral water bottles, Sprite bottles, coffee cups, black takeaway containers, and nonwoven fabric materials (**Figure** **4**a). Their performance within the photothermal catalytic system was systematically investigated. As shown in Figure 4b, the nonwoven fabric exhibited the highest specific activity (216.9  mg_BHET_·mg_cat_^−1^·h^−1^), which is 2.2 to 5.3 times higher than that of the bottle‐type materials (ranging from 40.8 to 98.0  mg_BHET_·mg_cat_^−1^·h^−1^). This substantial difference is mainly attributed to the surface area effect.^[^ ^31^ ^]^ Due to its micron‐scale fibrous structure, the nonwoven fabric has a significantly larger specific surface area than conventional bottle flakes, enhancing interfacial contact between the catalyst and substrate.

To further evaluate the feasibility and scalability of this system under realistic operating conditions, the research team designed and constructed an outdoor photothermal catalytic prototype device (Figure 4c). The system incorporates a 1.2‐meter‐diameter polymethyl methacrylate (PMMA) Fresnel lens as the concentrating element, achieving a concentration ratio of 120:1 and a high transmittance of 92% in the visible to near‐infrared range (400–1100  nm). Under actual sunlight conditions at 12:00 PM on April 3, 2025, in Suzhou Industrial Park (latitude 31.3° N), the system achieved a peak focused light intensity of 0.71 W·cm^−2^. Starting from an ambient temperature of 10 °C, a 100 mL graphite–ethylene glycol suspension was rapidly heated to 200 °C within 20 minutes, corresponding to a heating rate of 9.5 °C min^−1^—demonstrating excellent photothermal conversion efficiency.

Using this prototype, a photothermal catalytic depolymerization experiment was conducted with 20  g of real PET waste (approximate thickness: 20  µm; individual flake area < 1  cm^2^). Complete depolymerization was achieved in just 15 minutes (Figure 4d). Subsequent hot filtration (>90 °C) effectively removed residual PET and graphite, yielding a conversion rate exceeding 95%. After cooling the solution to 4 °C, BHET was initially precipitated and further purified via aqueous recrystallization, producing 17.1  g of high‐purity BHET and an overall yield of 64.6% (Figure 4e). The ^1^H NMR spectrum (Figure 4f) revealed characteristic peaks at δ = 8.13  ppm (aromatic protons), δ = 4.33  ppm (–CH_2_O–), and δ = 3.72  ppm (–OCH_2_CH_2_O–), consistent with literature‐reported spectra of BHET. These results confirm the high structural purity of the product and underscore the feasibility of closed‐loop chemical recycling under practical conditions.

The recyclability and retention of catalytic activity are critical indicators for evaluating the industrial feasibility of a catalyst. As shown in Figure S21 (Supporting Information), the recovered EG solution exhibited almost no PET depolymerization activity when no fresh anode was added. This indicates that the deprotonated EG (C_2_H_4_O_2_Li_2‐x_H_x_) generated in the previous reaction was consumed during catalysis, resulting in the loss of activity. Notably, when fresh graphite anode containing active lithium was added to the recovered EG solution, the catalytic activity was fully restored to its initial level, with PET conversion and BHET yield comparable to those of the first cycle.

### 2.4 Technical‐Economic Analysis

**Figure 5:**
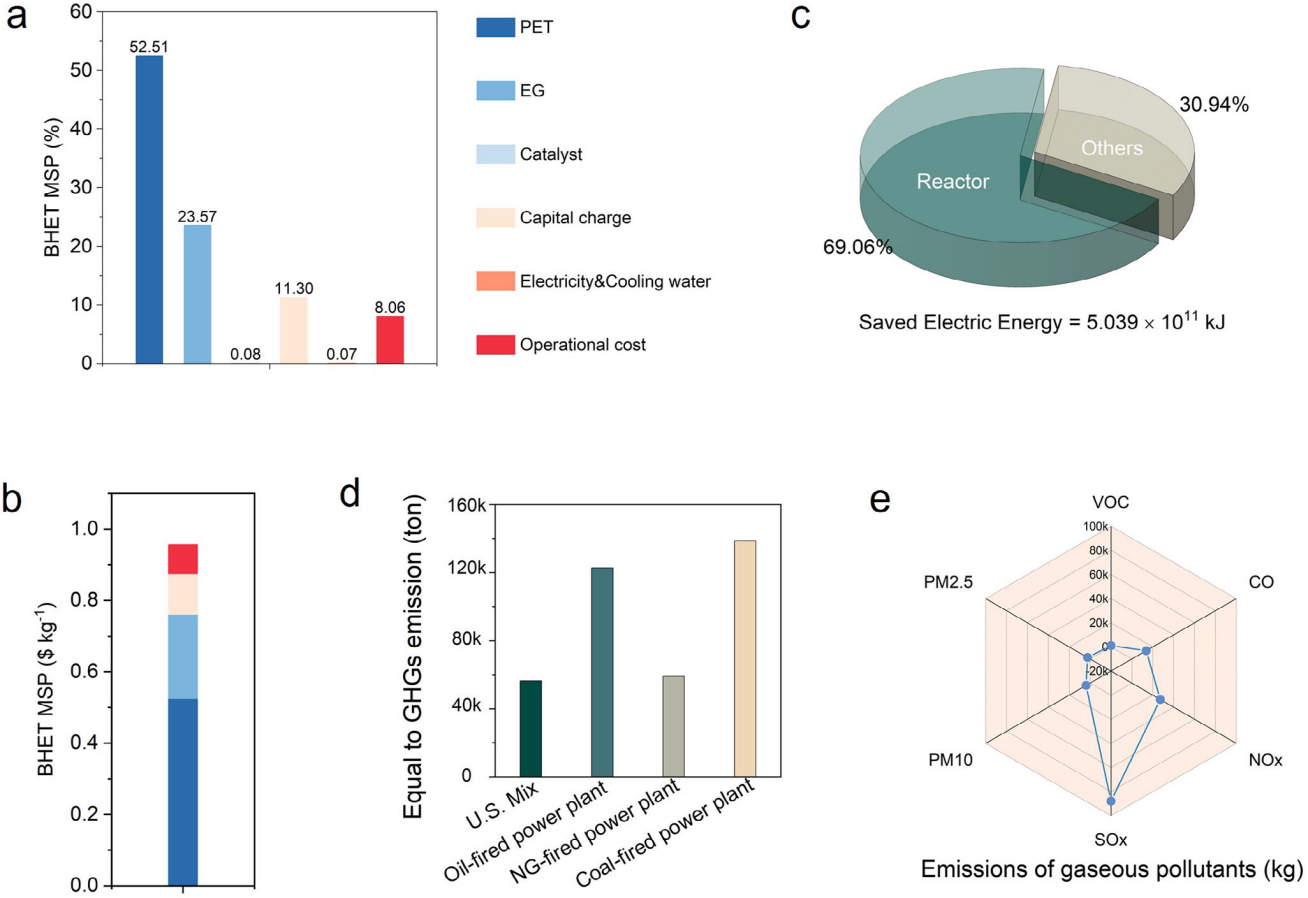
Economic impacts of photothermal catalysis. a) The percentage contribution of each factor to BHET MSP. b) BHET MSP of each factor. c) Simplified estimation of energy consumption in different equipment for recycling 90 000 tons of PET. d) Impact of photothermal catalysis on reducing greenhouse gas (GHG) emissions. e) Emissions of gaseous pollutants compared to conventional thermal catalysis.

In this study, we established a process flow model for a PET depolymerization plant with an annual capacity of 90000 tons using Aspen Plus V12, simulating BHET monomer production via a photothermal catalytic system based on organolithium compounds (C_2_H_4_O_2_Li_2‐x_H_x_). A Discounted Cash Flow (DCF) analysis was employed to evaluate the economic feasibility and determine the Minimum Selling Price (MSP) of BHET. The model assumes a PET waste procurement cost of $0.66·kg^−1^, EG at $0.96·kg^−1^, and the catalyst C_2_H_4_O_2_Li_2‐x_H_x_ at $0.25·kg^−1^. A single‐use scenario was adopted to reflect a conservative estimate. Under these assumptions, the calculated MSP of BHET was $0.956·kg^−1^. Cost structure analysis (**Figure** **5**a,b) indicates that PET feedstock accounts for the largest portion of the total cost (52.5%), followed by EG (23.6%). In contrast, the catalyst contributes only 0.08%, demonstrating excellent economic compatibility while maintaining high catalytic activity. Even under non‐recyclable conditions, the catalyst imposes minimal cost pressure, supporting its industrial applicability.

In terms of energy efficiency, the photothermal catalytic process offers substantial energy savings over conventional electrically heated thermal catalysis. It is estimated to reduce electricity consumption by approximately 5.039 × 10^11^ kJ per year (Figure 5c), with the reactor section alone contributing 69.1% of the total savings—highlighting the core advantage of solar‐driven catalysis in energy and cost reduction. When integrated with regional solar or low‐carbon electricity infrastructures (e.g., the U.S. grid mix), further environmental benefits can be achieved. To assess the environmental impact, we conducted a GHG emission analysis (Figure 5d). Results show that the photothermal system can reduce annual CO_2_‐equivalent emissions by ≈56 401.5 tons, significantly outperforming conventional coal‐powered thermal systems. Additionally, analysis of acidic gas emissions (Figure 5e) indicates an annual reduction of ≈136 348.2 tons of atmospheric pollutants—including volatile organic compounds (VOCs), carbon monoxide (CO), nitrogen oxides (NO_x_), sulfur oxides (SO_x_), and particulate matter (PM_10_/PM_2.5_). Notably, SO_x_ was the dominant pollutant, suggesting that integrated flue gas treatment and desulfurization technologies should be considered during system scale‐up to ensure compliance with environmental regulations.

## 3 Conclusion

In this study, we developed a cross‐disciplinary recycling strategy that bridges battery and plastic waste valorization via photothermal catalysis. By harnessing the inherent reactivity of spent graphite anodes and SEI‐derived species, we constructed a catalyst system capable of rapid PET depolymerization under solar irradiation. This approach not only improves the material utilization efficiency of low‐value LiFePO_4_ battery components but also offers a practical solution to polyester plastic pollution. Beyond its catalytic performance, the process demonstrates strong industrial potential, owing to its operational simplicity, low material cost, and compatibility with existing infrastructure. The simultaneous recovery of copper and graphite further enhances resource circularity. Looking forward, several challenges remain to be addressed to realize full‐scale implementation. These include elucidating the precise catalytic roles of SEI‐derived lithium salts, improving catalyst recyclability and regeneration protocols, and evaluating the adaptability of the system to diverse spent anode compositions.

Importantly, the electrolyte plays a decisive role in Li intercalation/deintercalation, governing the structural integrity and reversibility of lithiated graphite.^[^ ^32^, ^33^ ^]^ By regulating SEI formation and Li⁺ solvation, compatible electrolytes ensure efficient LiC_6_ formation, thereby impacting both battery performance and the recyclability of spent graphite.^[^ ^34^, ^35^ ^]^ Building on this mechanistic understanding, future efforts should clarify the catalytic roles of SEI‐derived lithium salts, improve catalyst recyclability and regeneration protocols, and evaluate system adaptability to diverse anode chemistries, paving the way toward scalable implementation.

## 4 Experimental Section

### Catalyst Preparation

The waste cylindrical LFP batteries were visually inspected to select those without obvious damage or leakage. Then, they were discharged at a constant current of 1C down to 2.5 V to ensure the safety and accuracy of subsequent charging experiments. The pretreated waste cylindrical LFP batteries were charged using a constant current–constant voltage (CC–CV) mode with a cutoff voltage of 3.8 V, a charging rate of 0.1 C, and a cutoff current rate of 0.01 C. After charging, the waste batteries were disassembled inside a glovebox to separate the anode from other battery components. The obtained anode was washed three times with DMC to remove residual electrolyte. The spent graphite anode was placed in a sealed glass vessel fitted with a rubber stopper, and immediately immersed in a predetermined volume of EG upon removal from the glovebox. Active lithium reacted slowly with ethylene glycol, releasing hydrogen gas throughout the 6‐hour reaction. After the reaction, the graphite spontaneously peeled off from the copper current collector. The copper foil was removed, and the solution was then sonicated for 10 min to further dissolve the residual lithium remaining in the waste graphite.

### Photothermal Catalytic Experiment for PET Glycolysis

Commercial PET film (thickness: 20  µm) and ethylene glycol (EG) were purchased from Alibaba and Sigma‐Aldrich, respectively. Prior to use, the PET film was cut into flakes with an area of less than 1  cm^2^. For a typical experiment, 2.5  mL of the pre‐prepared catalyst solution was added to a quartz reactor, followed by 0.5  g of PET flakes. The reactor was then sealed. Photothermal depolymerization was carried out under xenon lamp irradiation with a simulated solar intensity, and the reaction temperature was monitored using a thermocouple. For comparison, thermal depolymerization experiments were conducted in an oil bath under identical conditions (temperature and reaction time). After the reaction, PET, graphite, and the BHET product were separated by hot filtration and recrystallization. Specifically, 30  mL of deionized water was added to the reactor and heated to boiling. The hot mixture was immediately filtered to remove insoluble residues composed mainly of unreacted PET, oligomers, and graphite. The residue was dried at 80  °C for 24  h and weighed to calculate the solid‐phase conversion. The filtrate, containing EG, BHET, and water, was stored at 4 °C for 24  h to induce crystallization of BHET. The crystals were recovered by a second filtration, dried at 60  °C for 10  h, and weighed to determine the BHET yield.^[^ ^36^, ^37^ ^]^

## Conflict of Interest

The authors declare no conflict of interest.

## Author Contributions

Y.X. and M.Q. contributed equally to this work. J.C. conceptualized the idea for the study. Y.X., M.Q., and B.J. performed the investigation. Y.X., M.Q., P.X., and M.C. designed the methodology. Y.X. and J.C. wrote the original draft. J.C., P.X., and Q.Z. wrote, reviewed, and edited the manuscript. J.C. P.X. and Y.X. performed funding acquisition. J.C. and P.X. performed supervision.

## Supporting information



Supporting Information

## Data Availability

The data that support the findings of this study are available from the corresponding author upon reasonable request.
