# Supplementary material for: Upcycling Spent Graphite Anodes into Bifunctional Photothermal Catalysts for Efficient PET Chemical Recycling
Source: Adv Sci (Weinh). 2025 Sep 17;12(45):e10772. doi: 10.1002/advs.202510772 (PMC12677645; doi:10.1002/advs.202510772)
Supplement: Supplementary file 1 — Supporting Information [file ADVS-12-e10772-s001.docx]

Supporting Information

Upcycling Spent Graphite Anodes into Bifunctional Photothermal Catalysts for Efficient PET Chemical Recycling

*Yeping Xie, Mingle Qiu, Binglei Jiao, Panpan Xu^*^, Muhan Cao, Qiao Zhang^*^, Jinxing Chen^*^*

Y. Xie, M. Qiu, B. Jiao, Q. Zhang, J. Chen

State Key Laboratory of Bioinspired Interfacial Materials Science, Institute of Functional Nano & Soft Materials (FUNSOM), Soochow University, Suzhou 215123, PR China
E-mail: [chenjinxing@suda.edu.cn](mailto:chenjinxing@suda.edu.cn) (J.C.)

Y. Xie, M. Qiu, B. Jiao, M. Cao

Institute of Functional Nano & Soft Materials (FUNSOM), Jiangsu Key Laboratory of Advanced Negative Carbon Technologies, Soochow University, Suzhou 215123, P. R. China

1. Xu

Soochow Institute for Energy and Materials Innovations, College of Energy, Soochow University, 215006, Suzhou, P. R. China

**Characterization**

The surface morphology of the graphite anode was characterized using field-emission scanning electron microscopy (FE-SEM, TECNAI G2 F20, FEI, USA). The crystal structure and its evolution upon air exposure were analyzed by X-ray diffraction (XRD, Empyrean, PANalytical B.V., Netherlands) using Cu Kα radiation (λ = 1.5406 Å) at an accelerating voltage of 40 kV and a current of 40 mA. The diffraction patterns were recorded over a 2θ range of 10–90° at a scanning rate of 5°/min. The particle size distribution and zeta potential of the graphite were measured using a laser particle size and zeta potential analyzer (Nano ZS90, Malvern Instruments, UK). The molecular structure of the reaction products was confirmed by nuclear magnetic resonance (NMR) spectroscopy (AVANCE NEO 400, Bruker, Germany). The total lithium content was quantified by inductively coupled plasma optical emission spectrometry (ICP-OES, Varian 710-ES, USA). X-ray photoelectron spectroscopy (XPS) analysis was carried out using a Shimadzu ULTRADLD instrument (Shimadzu, Japan). The hydrogen evolution during the reaction was measured using a gas chromatograph (GC7900, Tianmei, China).

**Supplementary Figures**


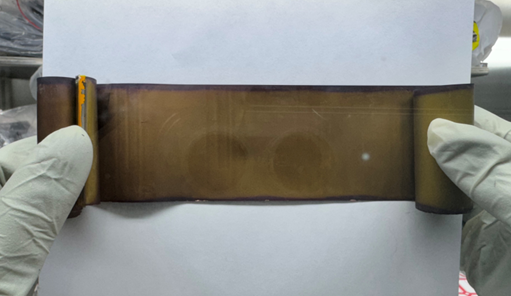


**Figure S1.** Unfolded graphite anode.


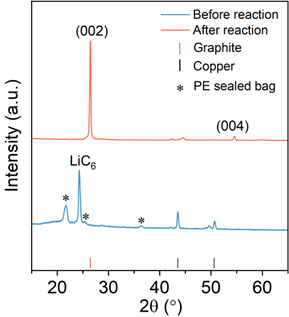


**Figure S2.** The X-ray diffraction (XRD) patterns of lithiated graphite (Placed in a polyethylene sealed bag) and graphite after reaction with ethylene glycol and water washing.

Notably, prior to XRD analysis, the graphite anodes were sealed in polyethylene zip-lock bags inside the glovebox to effectively exclude oxygen during the measurement.


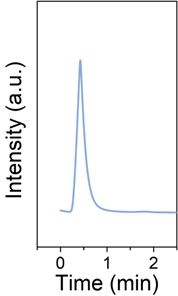


**Figure S3.** The obtained GC chromatogram.


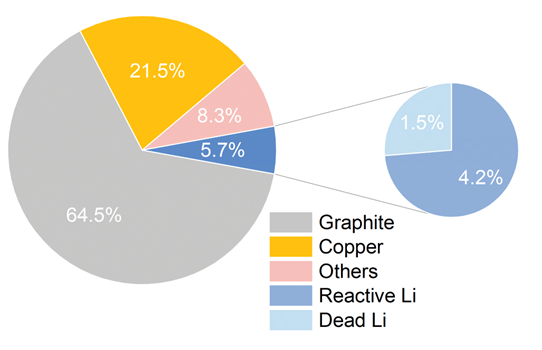


**Figure S4.** Mass fractions of each component in the anode.


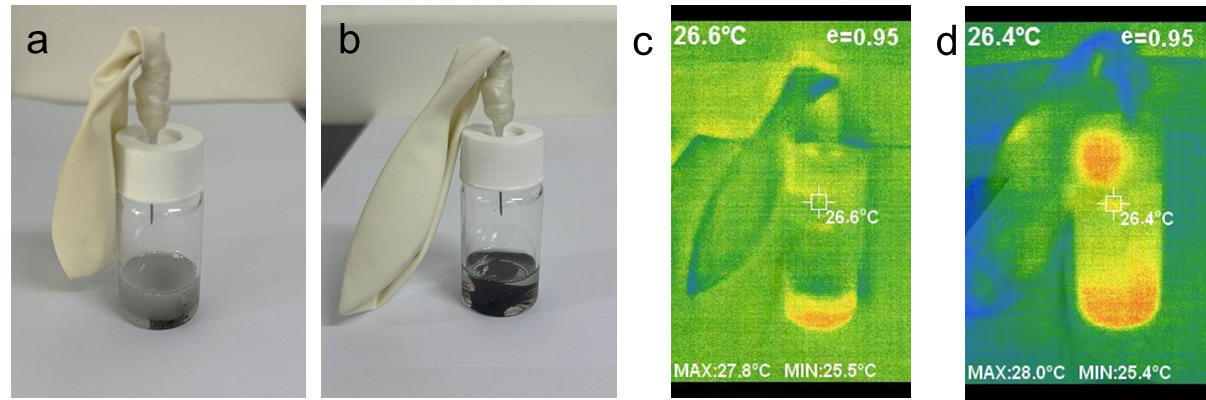


**Figure S5.** Electron images of the reaction between ethylene glycol and active lithium in the graphite anode, along with the heat evolution during the reaction. a, 0 min; b, 6 h; c, 5min; d, 1 h.

To verify the safety of the process, we monitored both the hydrogen evolution rate and the solution temperature during the reaction. All experiments were conducted in sealed 20 mL glass vials, using 60 mg of the graphite anode and 5 mL of ethylene glycol. The hydrogen generated during the reaction was initially collected in a balloon and subsequently flushed with nitrogen after the reaction to prevent contact with air, ensuring safe operation. Experimental results (**Figure S5**) showed that when ethylene glycol was used as the proton donor, the reaction commenced immediately upon addition, proceeding at a slow rate. Gas bubbles continued to evolve for approximately 6 hours before completely ceasing, and the final balloon volume exhibited only minor change, indicating a limited amount of hydrogen release. Graphite detachment from the copper foil was also observed. Infrared thermography recorded the temperature changes 5 minutes and 1 hour after ethylene glycol addition, revealing that the heat released during the reaction was very low. In summary, the hydrogen evolution in the ethylene glycol system is mild, and since the entire process occurs in a sealed environment, there is no risk of hydrogen accumulation or explosion. These results experimentally demonstrate the safety and feasibility of using the ethylene glycol system for the recovery of active lithium.

**Figure S6.** XRD patterns of graphite anode after reaction with ethylene glycol for different durations.

The reaction process of the graphite anode and ethylene glycol was monitored by XRD (**Figure S6**) : the graphite anode was rapidly placed in the XRD sample holder, five drops of EG solution were promptly added, and the measurement was initiated immediately. Upon contact with EG, the graphite anode reacted almost instantaneously—the golden surface of the anode sheet quickly turned black, accompanied by the evolution of gas bubbles. The initial XRD pattern exhibited a strong LiC_6_ diffraction peak along with weaker peaks of LiC_12_ and graphite. As the reaction proceeded, the LiC_6_ peak gradually decreased in intensity, while the LiC_12_ and graphite peaks became progressively stronger. After approximately 30 minutes, the LiC_6_ and LiC_12_ peaks had completely disappeared, leaving only the characteristic diffraction peaks of graphite. This evolution of diffraction signals clearly reveals the stepwise migration of lithium from between the graphite layers into the liquid phase, the restoration of the graphite interlayer spacing, and the underlying mechanism of lithium extraction and organolithium species formation.


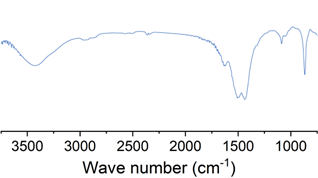


**Figure S7.** Infrared (IR) spectrum curve of the organolithium salt.


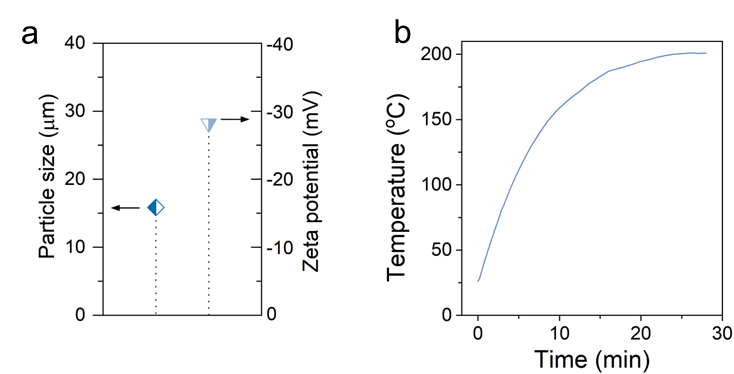


**Figure S8.** a) Laser particle size analysis results of graphite materials. b) Photothermal conversion performance curve of the graphite-containing ethylene glycol solution.

**Figure S9.** Effect of EG content on PET conversion and BHET yield under photothermal catalysis, reaction conditions: T = 180 ^o^C, mPET = 0.5 g, manode = 15 mg, t = 1.5 h.

The effect of EG dosage on PET conversion and BHET yield displayed distinct trends (**Figure S9**). Increasing the volume of EG led to a decrease in PET conversion, likely due to dilution of active species concentration, which slowed the depolymerization rate. The BHET yield reached a maximum at an EG dosage of 2.5 mL, indicating an optimal balance between reaction rate and product recovery. Insufficient EG resulted in a relative excess of basic species, promoting over-deprotonation of BHET and formation of lithium salts, which inhibited its precipitation during cold crystallization. Conversely, excessive EG reduced PET conversion, directly limiting BHET formation.


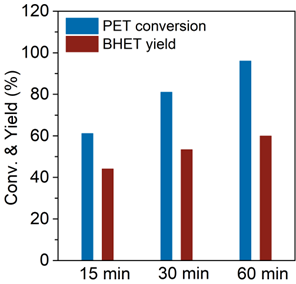


**Figure S10.** Effect of reaction time on PET conversion and BHET yield under photothermal catalysis, reaction conditions: T = 175 ^o^C, m_PET_ = 0.5 g, V_EG_ = 2.5 mL, m_anode_ = 50 mg.

**Figure S11.** Effect of reaction time on PET conversion and BHET yield under photothermal catalysis, reaction conditions: T = 180 ^o^C, m_PET_ = 0.5 g, V_EG_ = 2.5 mL, m_anode_ = 15 mg.

As shown in **Figure S11**, both PET conversion and BHET yield increased progressively with reaction time, demonstrating clear time-dependent behavior. Extending the reaction time to 2 hours resulted in approximately 90% PET conversion and over 70% BHET yield. However, to avoid unnecessary energy consumption and potential side reactions, excessively long reaction times should be avoided even when pursuing higher yields.


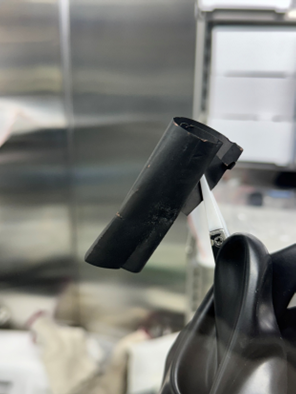


**Figure S12.** Unfolded discharged graphite anode.

As shown in **Figure S12**, the fully discharged graphite anode exhibits a typical black color, distinctly different from the golden-yellow appearance of lithiated graphite (LiC_6_). X-ray diffraction (XRD) analysis (**Figure S13**) shows no characteristic diffraction peaks of LiC_6_ or LiC_12_, but only the 002 and 004 peaks of graphite and peaks corresponding to the copper foil, indicating that the active lithium has been completely transferred to the cathode during discharge. Inductively coupled plasma atomic emission spectroscopy (ICP-AES) results reveal that the lithium content in the fully discharged anode is significantly reduced to approximately 1.27%, much lower than that in the charged graphite anode.

**Figure S13.** X-ray diffraction (XRD) patterns of discharged graphite anode samples placed in polyethylene-sealed bags under argon atmosphere and then exposed to air.

Notably, prior to XRD analysis, the graphite anodes were sealed in polyethylene zip-lock bags inside the glovebox to effectively exclude oxygen during the measurement.


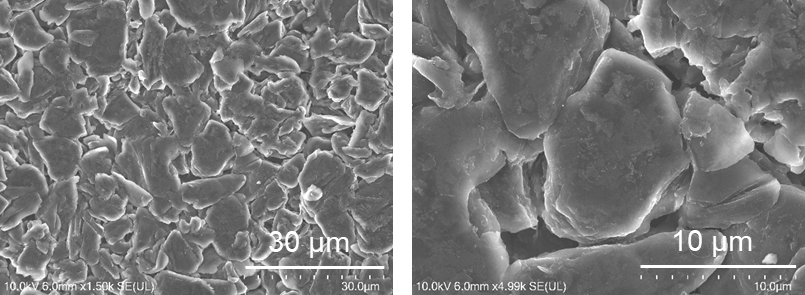


**Figure S14.** SEM images of the discharged graphite anode surface.

Scanning electron microscopy (SEM) images (**Figure S14**) show that the surface of the fully discharged anode is relatively smooth and lacks the morphological changes induced by lithiation. Elemental analysis (**Figure S15**) detected minor amounts of oxygen, fluorine, and phosphorus on the surface, which are likely residues of the solid electrolyte interphase (SEI) layer and its decomposition products.


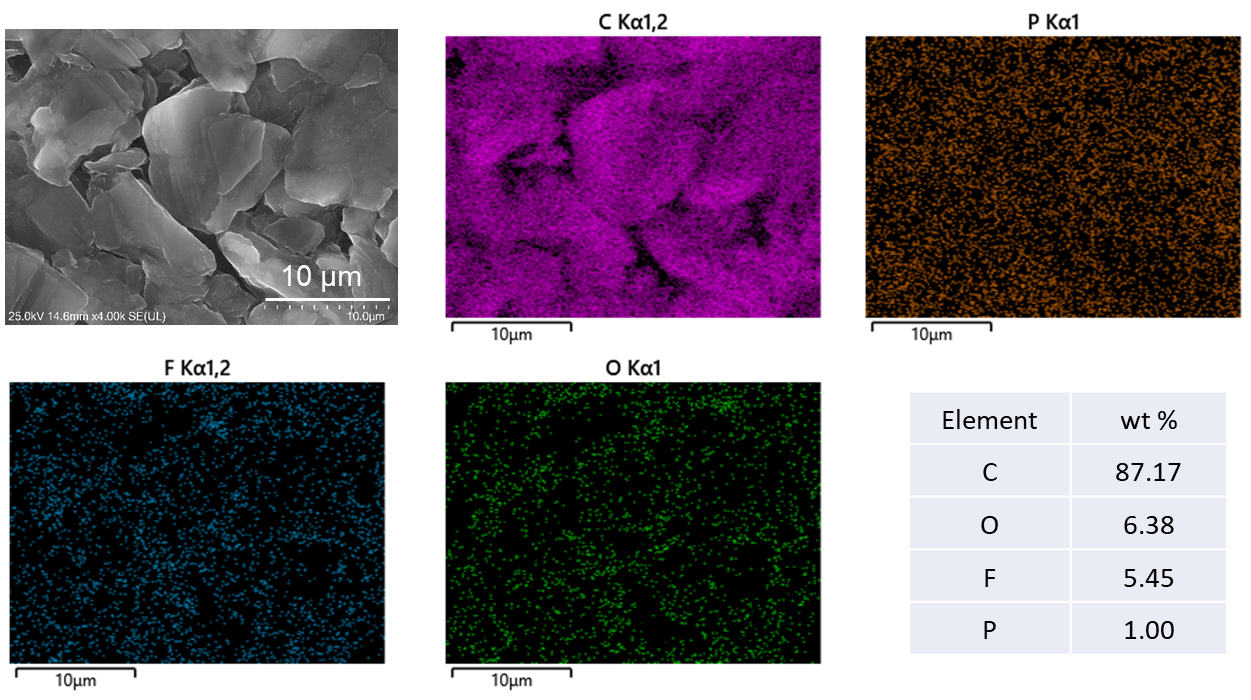


**Figure S15.** Elemental distribution mapping of the discharged graphite anode surface and element content.

Under identical conditions (anode loading of 50 mg and reaction time of 15 minutes), the fully discharged graphite anode exhibits negligible catalytic activity for PET depolymerization, with PET conversion below 5%. In contrast, the lithiated graphite (LiC_6_) anode shows significant catalytic performance, achieving PET conversion above 90%. These results clearly demonstrate that the catalytic activity primarily originates from LiC_6_ reacting with ethylene glycol to form alkoxide species, while the contributions from graphite defects and the SEI layer are comparatively minor.


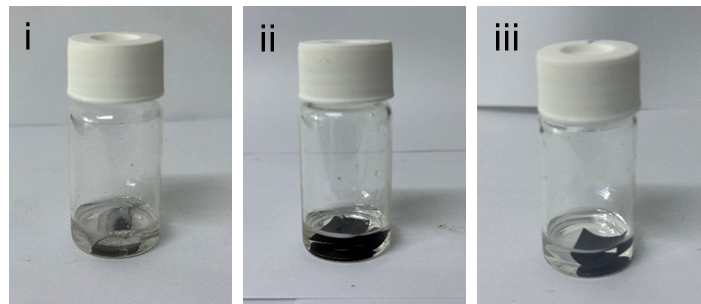


**Figure S16.** Digital photographs of graphite anodes after air exposure for varying durations and subsequent immersion in ethylene glycol. i, 10 minutes; ii, 1 h; iii, 24 h.

Regarding to the stability of LiC_6_, we have conducted additional experiments to examine the PET depolymerization performance of the catalyst after different air exposure durations (e.g., 10 min, 1 h, 24 h), in order to assess its stability and operational feasibility. Our results (**Figure S16**) show that the graphite anode exposed to air for 10 min could still react with EG, releasing hydrogen gas, indicating that reactive lithium species were still present. In contrast, no bubble formation was observed when samples exposed for 1 h or 24 h were immersed in EG, suggesting that the reactive lithium had been largely depleted. This observation is consistent with the XRD results in **Figure. 2b**, where LiC_6_ rapidly transforms into LiC_12_ upon air exposure and eventually converts completely to graphite.

**Figure S17.** Effect of exposure time on PET conversion and BHET yield under photothermal catalysis, reaction conditions: T = 180 ^o^C, m_PET_ = 0.5 g, V_EG_ = 2.5 mL, t = 15 min.

Catalytic performance evaluation (**Figure S17**) revealed that the EG solution prepared from the 10 min-exposed sample retained high catalytic activity for PET depolymerization (PET conversion ≈ 80%, BHET yield ≈ 45%). For the 1 h-exposed sample, the activity dropped significantly (PET conversion ≈ 55%, BHET yield ≈ 35%), while the 24 h-exposed sample exhibited a marked loss of activity (PET conversion < 10%). These results indicate that the catalyst tolerates short-term air exposure to some extent, but prolonged exposure substantially reduces its performance.


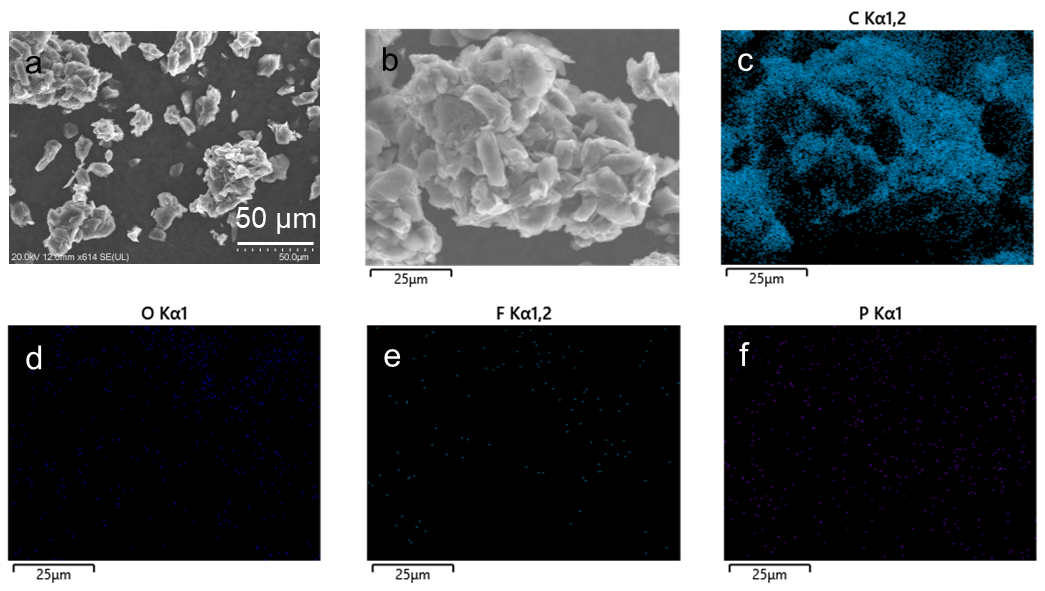


**Figure S18.** SEM images and elemental distribution on the surface of the graphite after reaction and water washing.


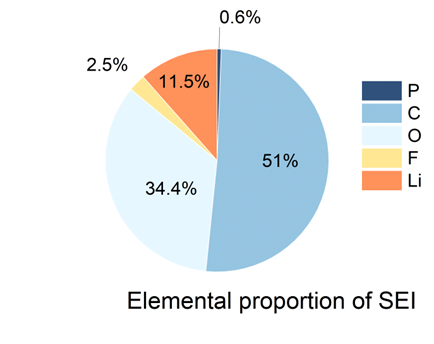


**Figure S19.** Elemental composition of the lithiated graphite surface.


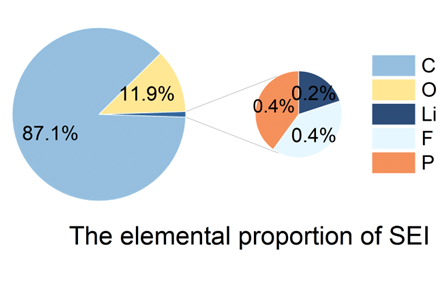


**Figure S20.** Elemental composition of the graphite after reaction and water washing.

**Figure S21.** catalyst recyclability, reaction conditions: T = 180 ^o^C, mPET = 0.5 g, V_EG_ = 2.5 mL, m_anode_ = 50 mg, t = 15 min.

To assess the stability of our system, we examined the performance of the graphite-anode–based photothermal catalyst over three consecutive reaction cycles. Specifically, after each reaction, the filtrate obtained from cold crystallization of BHET was concentrated under reduced pressure at 80 °C to remove water, and the recovered ethylene glycol was reused as the solvent for the next cycle.

**Table. S1** Yearly operating cost breakdown (base case).

**Basic PET 0.66 $ kg^–1^; EG 0.96 $ kg^–1^; EGLi 0.25 $ kg^–1^**

| Operating parameters | | | | | |
| --- | --- | --- | --- | --- | --- |
| Metric/Parameter | | Value | Units | | |
| Annual operating factor | | 3000 | hrs yr^–1^ | | |
| Feedstock contaminants | | 5% | Wt% | | |
| Feedstock PET | | 90000 | Ton yr^–1^ | | |
| Total BHET rate | | 113115.3 | Ton yr^–1^ | | |
| BHET yield | | 1.257 | Ton BHET (ton PET feed) ^–1^ | | |
| PET flake feed mass flow | | 30000 | Kg h^–1^ | | |
| BHET production rate | | 37705.1 | Kg h^–1^ | | |
| Variable Operating Costs | | | | | |
| Process hierarchy | Raw material/utility | | | Mass flow (kg h^–1^) | M$ yr^–1^ |
| Raw materials | | | | | |
| Feedstock pretreatment | PET flake feedstock | | | 30000 | 59.400 |
| PET depolymerization | EG | | | 9205 | 26.661 |
|  | EGLi | | | 126 | 0.095 |
| Clarification | Ultrafiltration unit replacement | | | - | 0.672 |
| Crystallization | Membrane replacement | | | - | 0.048 |
| OSBL Utilities | Cooling water | | | - | 0.048 |
|  | Grid electricity | | | - | 0.036 |
|  | Subtotal | | | - | 86.960 |
| Total variable operating cost | | | | | **86.960** |
| Fixed operating costs | | | | | |
| Labor & supervision | | | | | |
| Total salaries (managers, supervisors, engineers, technicians, administrative staff) | | | | | 0.810 |
| Labor burden (90% of total salaries) | | | | | 0.729 |
| Other overhead | | | | | |
| maintenance | | | | | 0.078 |
| Property insurance & tax | | | | | 6.779 |
| Total fixed operating costs | | | | | **8.396** |
| Total operating costs | | | | | **95.356** |

Tax **12.501** M$ yr^–1^ Capital Depreciation **0.285** M$ yr^–1^

All **108.142** M$ yr^–1^ MSP **1.202** $ kg^–1^

**Table. S2** Simplified breakdown of the minimum selling price of rBHET in the base case.

| **Cost Category** | **Cost Contribution ($ kg rBHET^–1^)** |
| --- | --- |
| PET | 0.5251 |
| EG | 0.2357 |
| EGLi | 0.0008 |
| Capital charge | 0.1130 |
| Electricity & Cooling water | 0.0007 |
| Operational cost | 0.0806 |
| **MSP** | **0.956** |
